# Parental opposition to comprehensive sexuality education in Australia: associations with religiosity and school sector

**DOI:** 10.3389/fpsyg.2024.1391197

**Published:** 2024-11-01

**Authors:** Jacqueline Hendriks, Neil Francis, Hanna Saltis, Katrina Marson, Jenny Walsh, Natasha Lawton, Sharyn Burns

**Affiliations:** ^1^Discipline of Health Promotion and Sexology, Curtin School of Population Health, Curtin University, Perth, WA, Australia; ^2^Collaboration for Evidence, Research and Impact in Public Health, Curtin University, Perth, WA, Australia; ^3^Law School, Swinburne University of Technology, Melbourne, VIC, Australia; ^4^The Hum Academy, Melbourne, VIC, Australia; ^5^Talk Revolution, Brisbane, QLD, Australia

**Keywords:** comprehensive sexuality education, parent attitudes, religion, religiosity, school sector, Australia

## Abstract

**Purpose:**

To empirically examine associations between parental opposition towards comprehensive sexuality education (CSE) and religiosity.

**Methods:**

A nationally representative survey of Australian parents (*N* = 2,418) examined opposition towards 40 CSE topics, by parental religiosity and secular/religious school sector.

**Results:**

Whilst opposition to most CSE topics correlated positively with religiosity, even amongst *very* religious parents, disapproval was minimal (2.8–31.2%; or 9.0–20.2% netted against non-religious parents). Parents with children enrolled in a Catholic school were less likely than secular-school parents to oppose CSE. Those with children at other-faith-schools were more likely to oppose CSE, but again disapproval was minimal (1.2–21.9%; or 1.3–9.4% netted against secular-school parents).

**Discussion:**

Only small minorities of *very* religious parents and parents with children in religious schools opposed the teaching of various CSE topics. Decision-makers should therefore be cautious about assuming that CSE delivery is not widely supported by particular families.

## 1 Introduction

The positive and protective benefits that result from comprehensive sexuality education (CSE) are well documented (UNESCO, 2018; Goldfarb and Lieberman, 2021). However, the provision of CSE within schools can be impacted by concern that parents, family members or carers (hereby referred to as parents) oppose the delivery of particular topics (Goldman, 2008; Marson, 2019). Furthermore, parental support and engagement are an integral component to the provision of CSE if an evidence-based whole-school approach is utilized (UNESCO, 2018; Goldfarb and Lieberman, 2021; WHO and UNESCO, 2021).

Within the Australian context, a recent nationwide survey reported significant parental endorsement for school-based provision of CSE (Hendriks et al., 2023). These findings align with an earlier systematic review, which also reported positive attitudes towards CSE across multiple countries (Kee-Jiar and Shih-Hui, 2020). Notwithstanding the emphatic support displayed by Australian parents, CSE provision within Australian schools is widely varied (Ezer et al., 2020). Although CSE implementation is impacted by a variety of factors, opposition is often attributed to the perceived religiosity of parents and has been stated as the reason “why they choose faith-based schools” in Australia (Parkinson, 2023). This may result in curtailment or purposive avoidance of topics in certain school programs, despite their inclusion in Australian school curricula (ACARA, 2023) and international guidelines (UNESCO, 2018).

The impact religion has on CSE provision has been explored in a variety of contexts. For example, Wareham (2022) recently presented three normative case studies from Wales to help illustrate the inherent problems that result when CSE provision is impacted by faith-based ‘carve-outs.’ In contrast, Sanjakdar (2018) draws upon interviews with secondary students in New Zealand and Australia, to argue for the value of including religion in CSE and its ability to develop critical perspectives.

Presently in Australia, vague curriculum guidance affords schools with great flexibility to avoid certain CSE issues (Ezer et al., 2018). Students report a prevailing deficit discourse and general dissatisfaction with current offerings (Ezer et al., 2019; Waling et al., 2021; Waling et al., 2020; Vrankovich et al., n.d.), and for particular sub-populations, their sexuality is often marginalized or ignored (Frawley and O’Shea, 2020; Senior et al., 2020; Mulholland et al., n.d.). Finally, the teaching workforce is often poorly prepared or supported to deliver this content, resulting in discomfort and low confidence levels (Hendriks et al., 2024; Ezer et al., 2021; Burns et al., 2023; O’Brien et al., 2020).

Therefore, to further strengthen the evidence base regarding religion and attitudes toward CSE provision in Australian schools, specific empirical investigations were conducted to examine associations between parental religiosity and opposition towards teaching CSE topics. Based on the national dataset of Australian parents, who shared their perspectives towards a wide array of CSE topics (Hendriks et al., 2023), we undertook targeted analyses to examine if levels of support were associated with either (a) the personal importance of religion to their daily life, or (b) the school sector in which they had enrolled their child(ren).

## 2 Methods

### 2.1 Study design and participants

Australian parents with children enrolled in a primary or secondary school completed an online survey (N = 2,418), with items based on a previous Canadian study (Wood et al., 2021). Additional methodology details and preliminary findings have been published previously (Hendriks et al., 2023; Hendriks et al., 2024), and the study was approved by the Curtin University Human Research Ethics Committee (HRE2021-0483).

### 2.2 Measures

Simple demographic data was obtained from all participants. Furthermore, three specific items from the broader survey were the focus of this current analysis:

- Support for specific CSE topics: Respondents indicated the earliest grade level at which 40 different CSE topics should first be taught within a school context. Those selecting *this topic should not be taught* (i.e., at any grade level) were considered to oppose CSE in some form.
- Religiosity: Respondents indicated the importance of religion to their everyday life: *not at all* (hereafter “non-religious”), *not very, somewhat, very*.
- School sector: “secular” means their child(ren) attended only a government or a non-faith school; “Catholic” means any child attending a Catholic school; “other-faith” means any child attending a religious (not Catholic) school.

### 2.3 Statistical procedure

Crosstabulation analyses were conducted using commercial statistical software, with statistical significance determined via Chi square tests and confirmed via manual analysis in Microsoft Excel. *I do not know/prefer not to answer* responses ranged from 1.1 to 5.4% of responses for all topics except “abstinence,” which was 8.7%, and all were excluded from analysis.

## 3 Results

### 3.1 General

Nearly two-thirds of parents (63.0%) supported the teaching all 40 CSE topics, with fewer than one in five (18.9%) opposed to one or two topics, and a similar proportion (18.1%) opposed to three or more topics.

There was no statistical association between the gender of a parent and their objection to any (one or more) CSE topics. Parents with children only at primary school were more likely to oppose teaching one (but not more) CSE topic than parents with any child at a secondary school, but the difference was small (15.9% versus 12.1%, *p* < 0.01).

### 3.2 Parent religiosity

Among the 2,304 parents who answered the religiosity question, 42.3% were non-religious, 18.9% not very religious, 19.9% somewhat religious, and 18.9% very religious. Of parents with a child at any religious school, 28.3% were non-religious, 24.1% not very religious, 24.9% somewhat religious, and 22.8% very religious.

Table 1 shows the prevalence of child enrolment in government/secular versus religious schools by parental religiosity. Non-religious parents were significantly less likely (19.1% less likely than very religious parents, *p* < 0.001) to have enrolled their child/ren in a religious school. Amongst more religious parents there were minorities of and no significant differences in religious school enrolment (45.2% not very religious, 44.3% somewhat religious, and 42.8% very religious). That is, non-religious parents were more likely to reject religious schools, but the likelihood of selecting a religious school amongst other parents did not correlate with greater religiosity.

**Table 1:** Child school enrolment sector by parental religiosity.

	School sector enrolment by parental religiosity (%)					Paired differences		
	All	Not at all	Not very	Some what	Very	Very – Not at all	Very – Not very	Very – Somewhat
No child in a religious school	64.5	76.3***	54.8***	55.7***	57.2***			
Any child in a religious school	35.5	23.7***	45.2***	44.3***	42.8***	19.1***	−2.4	−1.6

*p*-values: *** < 0.001. Some differences may not appear exact due to rounding.

Table 2 shows the prevalence of parental opposition to the 40 CSE topics by parental religiosity. Overall, *very* religious parents were the most likely to oppose topics. Amongst these parents, opposition to teaching topics at school generally ranged from 2.8% (communication skills) to less than one-third (31.2%, sexual pleasure), compared with 0.2% (communication skills) to 11.2% (sexual pleasure) amongst non-religious parents.

**Table 2:** Prevalence of parental opposition to teaching CSE topics at school, by parental religiosity.

	Prevalence of opposition by parental religiosity (%)					Paired difference
Schools should (not) teach…	All	Not at all	Not very	Some what	Very	Very – Not at all
Oppose no topics	63.2	66.9**	65.4	68.6**	46.9***	−20.0***
Oppose 1 or 2 topics	19.0	20.4	19.7	14.9*	19.5	−0.9
Oppose 3 or more topics	17.8	12.7***	14.9	16.6	33.6***	20.8 ***
Individual topics						
Self-esteem and personal development	1.0	0.2**	0.2	1.8	2.8	2.6
Communication skills	1.1	0.4*	0.7	1.1	2.8	2.4
Personal safety (e.g., abuse prevention)	1.3	0.4**	1.4	1.8	2.6**	2.2
The impact of peer pressure	1.4	0.3***	0.7	1.3	4.5***	4.2***
Changes associated with puberty (e.g., physical, biological, psychological, emotional, social)	1.5	0.7**	0.9	1.8	3.5***	2.8***
Decision making skills	1.5	0.8*	1.4	0.9	3.8***	2.9***
Supporting and helping peers	1.7	0.9*	0.9	1.1	4.7***	3.8***
Correct names for body parts, including genitals	1.9	0.7***	1.2	2.7	4.7***	3.9***
Bodily autonomy and personal boundaries (e.g., a child’s body belongs to them)	1.9	0.8**	1.4	2.2	4.5***	3.7***
Sexually transmitted infections (STIs), including HIV	1.9	0.6***	1.2	1.8	5.6***	5.0***
Reproduction	2.1	0.8***	1.6	1.6	6.1***	5.3***
Healthy and unhealthy relationships	2.3	1.0***	2.1	2.0	5.4***	4.4***
Sex and the law	2.9	1.2***	1.9	2.7	8.0***	6.9***
Non-violent conflict resolution in relationships	3.1	1.3***	3.3	2.7	7.4***	6.1***
Prevention of sexual exploitation	3.0	2.3	1.4*	2.2	6.7***	4.3***
Body image	3.1	2.2*	1.6	2.9	6.8***	4.6***
Sexual and gender-based violence/harassment, coercion	3.1	2.0*	1.9	2.0	7.8***	5.8***
Contraception	3.3	1.4***	2.3	2.0	10.0***	8.6***
Sexual consent (e.g., communicating about consent for any/all sexual activity)	3.3	1.6***	2.6	2.2	9.0***	7.4***
How to access sexual health and reproductive health services	3.7	0.9***	2.8	3.2	11.2***	10.3***
Dealing with pressure to be sexually active	3.8	1.9***	1.9*	3.6	10.3***	8.4***
Safer sex methods (e.g., condom use)	3.9	1.7***	2.3	2.9	11.8***	10.1***
Emotional components of sexual relationships	4.2	2.4***	2.8	2.3*	11.7***	9.2***
Common/"popular” language related to relationships and sexual health	4.3	2.5***	2.1*	2.9	12.0***	9.5***
How power differences such as age, gender, socioeconomic status, race, or unequal position (e.g., student/teacher, supervisor/employee) may impact relationships	4.4	3.1*	4.0	3.6	8.4***	5.3***
Understanding and appreciation of different cultural approaches to relationships and sexual health	5.1	4.6	3.8	3.4	9.2***	4.6***
Sexuality and communication technology (e.g., “sexting”)	5.1	2.5***	2.8*	4.2	14.3***	11.8***
Media literacy skills related to sexual content in advertising, TV, pornography, etc.	5.2	3.7**	3.6	4.7	10.8***	7.0***
Reasons to engage or not engage in sexual activity	5.4	3.0***	4.0	4.1	13.8***	10.9***
Sexual behavior (e.g., different types of sexual behavior such as kissing, intercourse)	5.6	3.5***	4.0	4.3	13.4***	9.9***
Sexual problems and concerns	5.6	3.4***	4.5	4.5	13.1***	9.7***
Attraction, love, and intimacy	5.7	3.9**	4.2	3.1**	13.9***	10.1***
Sexuality and disability (e.g., physical disabilities, developmental disabilities)	6.9	5.3*	4.0*	5.5	14.7***	9.5***
The influence of sexually explicit media (e.g., pornography)	7.3	5.0*	4.3**	6.9	15.8***	10.8***
Gender roles and stereotypes	8.0	6.6*	7.3	6.1	14.1***	7.5***
Sexual orientation	10.9	7.2***	8.6	8.7	23.9***	16.7***
Information about masturbation	13.1	8.7***	9.6*	11.1	28.9***	20.2***
Abstinence	14.5	19.8***	13.0	7.9***	10.9*	−9.0***
Gender identity (i.e., how a person identifies based on an internal sense of who they are, such as girl/woman, boy/man, non-binary, etc.)	14.2	10.3***	12.2	13.2	25.8***	15.5***
Sexual pleasure	16.3	11.2***	16.1	13.0*	31.2***	19.9***
*All four* top “very religious” objections (sexual orientation, gender identity, masturbation, sexual pleasure)	4.3	2.5***	2.5*	2.0**	12.4***	9.9***

*p*-values: * < 0.05, ** < 0.01, *** < 0.001. Some differences may not appear exact due to rounding.

Opposition to the topic of abstinence was notable (19.8% amongst non-religious, 10.9% amongst very religious) and requires further exploration. We postulate that there may have been measurement error in that some respondents may have thought this item referred to abstinence-only education. Of note, 8.7% selected *I do not know/prefer not to answer* for this item, when for most other items the percentage was well below 5.0%.

Very religious parents were most likely to oppose topics related to sexual pleasure (31.2% sexual pleasure, 28.9% information about masturbation), gender identity (25.8%), sexual orientation (23.9%), and the influence of sexually explicit media (e.g., pornography; 15.8%). Amongst the remaining topics, opposition was less than 15% and for 9/40 topics it was less than 5%.

The prevalence of opposition amongst *very* religious parents netted against non-religious parents — to adjust for non-religious opposition — was statistically significant for most topics (37/40 topics, each *p* < 0.001). This provides additional evidence that higher levels of religiosity are associated with opposition towards CSE. However, the magnitude of these significant differences was modest, from 2.8% (changes associated with puberty) to 20.2% (information about masturbation), each *p* < 0.001. The only topic where *very* religious parents were less (not more) likely to oppose was abstinence (−10.9%, p < 0.001), again suggesting that some non-religious parents may have interpreted the question as abstinence-*only* education.

### 3.3 School sector

Table 3 shows the prevalence of parental opposition to the 40 CSE topics by school sector. Across all three school sectors, majorities of parents opposed none of the 40 topics (63.6% of secular-school-only parents, 65.4% of Catholic-school parents and 56.0% of other faith-school parents).

**Table 3:** Prevalence of parental opposition to teaching CSE topics at school, by child enrolment school sector.

	Prevalence of opposition by school sector (%)				Paired difference from secular-only	
Schools should (not) teach…	All schools	Secular only^a^	Any Catholic	Any other faith	Any Catholic	Any other faith
Oppose no topics	63.0	63.6	65.4	56.0**	1.7	−7.7**
Oppose 1 or 2 topics	18.9	19.2	18.4	18.5	−0.9	−0.8
Oppose 3 or more topics	18.1	17.1	16.3	25.6***	−0.9	8.5***
Individual topics						
Self-esteem and personal development	1.1	1.0	0.6	2.1	−0.5	1.1
Communication skills	1.1	1.1	1.0	1.2	−0.2	0.1
Personal safety (e.g., abuse prevention)	1.3	1.0*	2.3*	1.8	1.3	0.8
The impact of peer pressure	1.4	1.4	1.3	2.4	0.0	1.1
Changes associated with puberty (e.g., physical, biological, psychological, emotional, social)	1.5	1.5	0.8	2.7	−0.8*	1.2
Decision making skills	1.5	1.6	1.2	2.7	−0.4	1.2
Supporting and helping peers	1.7	1.8	1.2	2.1	−0.7	0.3
Correct names for body parts, including genitals	1.9	1.7	1.9	3.6*	0.2	1.9
Bodily autonomy and personal boundaries (e.g., a child’s body belongs to them)	1.9	2.0	1.2	3.0	−0.9	1.0
Sexually transmitted infections (STIs), including HIV	1.9	1.9	1.2	3.9**	−0.7	2.0*
Reproduction	2.2	2.3	1.3	3.3	−0.9	1.0
Healthy and unhealthy relationships	2.4	2.5	1.5	3.7	−1.0	1.1
Sex and the law	2.8	2.7	2.3	4.8*	−0.4	2.1*
Non-violent conflict resolution in relationships	3.0	2.9	3.3	3.7	0.4	0.8
Prevention of sexual exploitation	3.0	3.2	2.9	2.5	−0.3	−0.8
Body image	3.2	3.2	2.7	3.9	−0.5	0.8
Sexual and gender-based violence/harassment, coercion	3.2	3.7	1.5*	4.3	−2.1*	0.7
Contraception	3.3	3.3	1.9*	5.5*	−1.4	2.1
Sexual consent (e.g., communicating about consent for any/all sexual activity)	3.3	3.5	1.9*	4.6	−1.6	1.1
How to access sexual health and reproductive health services	3.6	3.5	2.3	5.8*	−1.2	2.2
Dealing with pressure to be sexually active	3.9	4.3	2.3*	5.8	−2.0*	1.6
Safer sex methods (e.g., condom use)	4.0	4.1	2.1*	7.0**	−2.0	2.9*
Emotional components of sexual relationships	4.3	4.5	2.5*	6.4*	−2.0*	1.9
Common/"popular” language related to relationships and sexual health	4.4	4.2	2.9	7.7**	−1.3	3.5**
How power differences such as age, gender, socioeconomic status, race, or unequal position (e.g., student/teacher, supervisor/employee) may impact relationships	4.4	4.0	4.5	7.1*	0.5	3.1*
Understanding and appreciation of different cultural approaches to relationships and sexual health	5.1	4.9	4.0	8.2**	−0.8	3.4*
Sexuality and communication technology (e.g., “sexting”)	5.2	5.7	3.1*	5.7	−2.7*	0.0
Media literacy skills related to sexual content in advertising, TV, pornography, etc.	5.3	5.2	4.3	6.7	−1.0	1.5
Reasons to engage or not engage in sexual activity	5.5	6.1	2.9**	7.0	−3.2**	0.9
Sexual behavior (e.g., different types of sexual behavior such as kissing, intercourse)	5.7	5.8	4.3	8.3*	−1.5	2.5
Sexual problems and concerns	5.7	5.7	3.8*	8.2*	−1.9	2.5
Attraction, love, and intimacy	5.7	5.5	5.4	7.6	−0.2	2.1
Sexuality and disability (e.g., physical disabilities, developmental disabilities)	7.0	7.4	5.3	7.8	−2.1	0.4
The influence of sexually explicit media (e.g., pornography)	7.4	8.4*	4.6**	7.1	−3.8**	−1.3
Gender roles and stereotypes	8.1	7.8	7.4	10.3	−0.4	2.6
Sexual orientation	11.2	10.1*	10.6	16.9***	0.5	6.8***
Information about masturbation	13.2	12.7	11.6	18.3**	−1.1	5.6**
Abstinence	14.2	14.1	12.8	16.7	−1.4	2.6
Gender identity (i.e., how a person identifies based on an internal sense of who they are, such as girl/woman, boy/man, non-binary, etc.)	14.3	12.6**	14.4	21.9***	1.8	9.4***
Sexual pleasure	16.6	16.4	14.3	20.6*	−2.1	4.2
*All four* top “very religious” objections (sexual orientation, gender identity, masturbation, sexual pleasure)	4.3	3.8	3.6	7.7***	−0.2	3.9***

^a^ Child(ren) enrolled only in a government or independent secular school. *p*-values: * < 0.05, ** < 0.01, *** < 0.001. Some differences may not appear exact due to rounding.

In comparison to secular-school parents, Catholic-school parents were often *less* likely to oppose CSE topics. However, net differences were only statistically significant for 7/40 topics. In comparison to secular-school parents, other-faith-school parents were often *more* likely to oppose CSE topics. Similarly, net differences were only statistically significant for 9/40 topics.

Amongst Catholic-school parents, opposition was 5% or less for 32/40 CSE topics. This included low levels of opposition towards topics such as contraception (1.9%) and safer sex methods (e.g., condoms; 2.1%) that are often considered contrary to a Catholic school education.

Opposition was more prevalent amongst other-faith-school parents, however, still at 5% or less for 18/40 topics. Amongst parents with children enrolled in a non-secular school, the greatest opposition was reserved for gender identity (14.4% amongst Catholic-school parents; 21.9% amongst other-faith-school parents) and sexual pleasure (14.3% amongst Catholic -school parents; 20.6% amongst other-faith-school parents).

### 3.4 Religious parents and school sector choice

To determine whether attitudes differed amongst religious parents with children in religious schools versus non-religious schools, the prevalence of opposition to CSE topics amongst *very* religious parents was compared by those with children only at non-religious schools, versus those with any child at a religious school (Table 4).

**Table 4:** Prevalence of opposition to CSE topics amongst very religious parents by child enrolment sector.

	Prevalence of opposition amongst *very* religious parents, by child enrolment sector (%)			
Schools should (not) teach…	All schools	No religious school	Any religious school	Difference: Any – No religious
Oppose no topics	46.9	47.0	46.8	−0.2
Oppose 1 or 2 topics	19.5	20.9	17.7	−3.1
Oppose 3 or more topics	33.6	32.1	35.5	3.4
Individual topics				
Personal safety (e.g., abuse prevention)	2.6	1.6	3.8	2.2
Self-esteem and personal development	2.8	3.7	1.7	−2.0
Communication skills	2.8	3.7	1.6	−2.1
Changes associated with puberty (e.g., physical, biological, psychological, emotional, social)	3.5	4.1	2.7	−1.4
Decision making skills	3.8	3.7	3.9	0.2
Bodily autonomy and personal boundaries (e.g., a child’s body belongs to them)	4.5	4.6	4.4	−0.1
The impact of peer pressure	4.5	4.2	5.0	0.9
Correct names for body parts, including genitals	4.7	3.7	6.0	2.3
Supporting and helping peers	4.7	5.3	3.9	−1.4
Healthy and unhealthy relationships	5.4	6.1	4.5	−1.7
Sexually transmitted infections (STIs), including HIV	5.6	5.3	6.0	0.7
Reproduction	6.1	5.4	7.1	1.8
Prevention of sexual exploitation	6.7	7.0	6.2	−0.9
Body image	6.8	8.2	4.9	−3.3
Non-violent conflict resolution in relationships	7.4	7.5	7.3	−0.2
Sexual and gender-based violence/harassment, coercion	7.8	8.6	6.7	−1.9
Sex and the law	8.0	8.3	7.7	−0.5
How power differences such as age, gender, socioeconomic status, race, or unequal position (e.g., student/teacher, supervisor/employee) may impact relationships	8.4	7.1	10.2	3.1
Sexual consent (e.g., communicating about consent for any/all sexual activity)	9.0	9.5	8.3	−1.2
Understanding and appreciation of different cultural approaches to relationships and sexual health	9.2	7.9	11.1	3.1
Contraception	10.0	10.0	10.0	0.0
Dealing with pressure to be sexually active	10.3	11.7	8.4	−3.3
Media literacy skills related to sexual content in advertising, TV, pornography, etc.	10.8	10.9	10.6	−0.3
Abstinence	10.9	9.1	13.1	4.1
How to access sexual health and reproductive health services	11.2	11.7	10.5	−1.2
Emotional components of sexual relationships	11.7	12.6	10.5	−2.1
Safer sex methods (e.g., condom use)	11.8	10.9	12.9	2.0
Common/"popular” language related to relationships and sexual health	12.0	11.3	12.9	1.6
Sexual problems and concerns	13.1	14.5	11.1	−3.4
Sexual behavior (e.g., different types of sexual behavior such as kissing, intercourse)	13.4	14.9	11.2	−3.7
Reasons to engage or not engage in sexual activity	13.8	16.3	10.6	−5.7
Attraction, love, and intimacy	13.9	14.8	12.8	−2.0
Gender roles and stereotypes	14.1	13.8	14.6	0.9
Sexuality and communication technology (e.g., “sexting”)	14.3	17.5	9.9	−7.6*
Sexuality and disability (e.g., physical disabilities, developmental disabilities)	14.7	15.6	13.5	−2.2
The influence of sexually explicit media (e.g., pornography)	15.8	18.1	12.8	−5.3
Sexual orientation	23.9	21.7	27.0	5.3
Gender identity (i.e., how a person identifies based on an internal sense of who they are, such as girl/woman, boy/man, non-binary, etc.)	25.8	21.5	31.7	10.2*
Information about masturbation	28.9	28.3	29.7	1.3
Sexual pleasure	31.2	31.7	30.5	−1.1
*All four* top “very religious” objections (sexual orientation, gender identity, masturbation, sexual pleasure)	12.4	10.8	14.5	3.7

*p*-values: * < 0.05. Some differences may not appear exact due to rounding.

Of the 40 topics, *very* religious parents with a child at a religious school appeared more likely to oppose 16 topics, but more likely to support 24 topics. However, the differences were small and only two were statistically significant. *Very* religious parents with a child at a religious school were 7.6 percentage points *less* likely (a difference of around one in 13 parents) to oppose schools teaching children about sexuality and communications technology (e.g., “sexting”) (*p* < 0.05), and 10.2 percentage points *more* likely (around one in 10 parents) to oppose teaching gender identity (*p* < 0.05).

Even amongst *very* religious parents with a child at a religious school, opposition to CSE topics was less than one third (maximum 31.7%, gender identity) and often much less.

## 4 Discussion

Although other studies in the previous decade have examined parental attitudes towards CSE, despite collecting data about the religious affiliations of their respondents, most have not factored this into their statistical analyses (Wood et al., 2021; Dake et al., 2014; Fisher et al., 2015; Kantor and Levitz, 2017; McKay et al., 2014). An exception has been the recent work of Hurst et al. (2024) who reported, based on a national sample of parents across the United States of America, that there was strong support for students to receive CSE focused on three content areas: factual knowledge, practical skills, and pleasure and identity. However, politically conservative parents who also expressed high levels of religiosity expressed lower levels of support (Hurst et al., 2024).

In this study, whilst parental opposition towards schools delivering various CSE topics is positively associated with religiosity, the magnitude of any dissent is modest. Even amongst Australian parents who are *very* religious, opposition towards CSE topics is less than one-third (maximum 31.2%) and considerably lower in most instances. Amongst all parents with children enrolled in religious schools (of which 22.8% are *very* religious and 28.3% are not at all religious), the level of opposition towards various CSE topics is an even smaller minority: a maximum of 14.4% amongst Catholic-school parents and 21.9% amongst other-faith-school parents. Compared with secular-school parents, Catholic-school parents are overall *less* likely to oppose topics, and the premium in opposition amongst other-faith-school parents is less than one-in-ten (up to 9.4%). At Australian religious schools, only minorities of all parents (maximum 21.9%) and even *very* religious parents (maximum 31.7%) oppose any CSE topic, and the prevalence of opposition is often much less. The contention that most or all religious-school parents, including *very* religious ones, oppose schools teaching CSE topics including the most contended topics of sexual orientation, gender identity and sexual pleasure, is rejected.

Given the minority prevalence of opposition to CSE topics amongst parents at Australian religious schools, the contention that most or even a majority choose religious schools significantly because of conservative or tradition-normative views regarding sexuality, is rejected.

The finding that *very* religious parents are not more likely than not-very-religious parents to choose a religious school is also consistent with rejecting the contention. However, we were unable to correct for possible differences in socio-economic status amongst the cohorts in regard to the ability of families to afford non-government school fees, and so this finding requires further study.

The findings complement other analyses our team has undertaken to demonstrate that most Australian parents express supportive attitudes towards diverse sexual orientations, gender diversity, and actions to address homophobia and transphobia. Such support is expressed by parents of all religious affiliations and parents who have enrolled their child(ren) in a religious school (Hendriks et al., 2024). Similarly, other Australian research has also reported low levels of opposition (5.6%) towards relationships and sexuality education amongst parents of children attending a government school (Ullman et al., 2022). However, the religiosity of the parent was not considered in any of their statistical analyses and only one school sector was considered.

### 4.1 Strengths, limitations and future directions

The data presented here is a sub-set of a much broader series of analyses. Additional data and details about strengths and limitations of the broader study have been reported previously (Hendriks et al., 2023; Hendriks et al., 2024). Whilst the sample closely matched population estimates it may not be truly nationally representative based on particular demographic characteristics (Hendriks et al., 2023). The survey instrument did not collect data about socio-economic status and was conducted in the English language only. However, in relation to the survey items that were the focus of this manuscript, a large and diverse sample was obtained. A significant proportion of the respondents identified themselves as religious and reported a broad array of religious affiliations that align closely with recent census results (Australian Bureau of Statistics, 2021). The proportion of respondents from each school sector closely approximates population estimates (Hendriks et al., 2024).

Our study does not provide evidence of attitudes at individual schools or at schools of a particular religious tradition other than Catholic. Nevertheless, given overall that minorities of parents — even *very* religious ones — at religious schools oppose CSE topics, an individual school with a majority of parents opposing a particular CSE topic may be possible. Schools should therefore be encouraged to engage widely and empirically with their parents to understand their viewpoints, rather than to make assumptions.

To further progress our understanding of the intersections between religiosity and CSE provision, future research should focus on qualitative data collection to provide greater insight into the perspectives of parents. Purposeful sampling frames should be used to ensure a diverse range of religious affiliations are captured, and parent perspectives should be triangulated with data from school students and teaching staff. Finally, future research and school programs should focus on trying to achieve pluralism in this space (Sanjakdar, 2018). Whilst we need to ensure particular viewpoints do not curtail evidence-based CSE provision, we similarly need to be respectful of religious perspectives.

## 5 Conclusion

These findings empirically dispute the contention that most parents who are *very* religious, or who have selected a non-secular school for their child(ren), do not endorse schools to deliver a comprehensive sexuality education program. Policymakers, educators, and other decision-makers should not assume the sexuality values held by parents. Furthermore, evidence-based guidelines direct that quality programs should embrace whole-school approaches that include strong parental engagement.

## Data Availability

The original contributions presented in the study are included in the article/supplementary material, further inquiries can be directed to the corresponding author.
